# Editorial: Roles and regulatory mechanisms of microRNA in plant development, evolution, and adaptation

**DOI:** 10.3389/fpls.2022.995517

**Published:** 2022-08-15

**Authors:** Xiaozeng Yang, Turgay Unver, Xiuren Zhang, Lei Li

**Affiliations:** ^1^Institute of Biotechnology, Beijing Academy of Agriculture and Forestry Sciences, Beijing, China; ^2^Beijing Key Laboratory of Agricultural Genetic Resources and Biotechnology, Beijing, China; ^3^Ficus Biotechnology, Ankara, Turkey; ^4^Department of Biochemistry and Biophysics, Texas A&M University, College Station, TX, United States; ^5^Department of Biology, Texas A&M University, College Station, TX, United States; ^6^State Key Laboratory of Protein and Plant Gene Research, Peking-Tsinghua Center for Life Sciences, School of Life Sciences and School of Advanced Agricultural Sciences, Peking University, Beijing, China

**Keywords:** microRNA, regulation, plant, morphology, stress, network

MicroRNAs (miRNAs) are a group of endogenous small non-coding RNAs that regulate gene expression. In general, miRNAs can guide their effector partners in the cytoplasm, Argonaute proteins, to silence transcripts with sequence complementarity by mRNA cleavage or translation resting. They can also control gene expression by targeting promoters or enhancers in the nucleus. In plants, miRNAs regulate plant development and response to environmental stresses by controlling expression levels and activities of a variety of downstream genes. Even though a large number of plant miRNAs have been identified and linked to various cellular processes, our understanding of the precise roles and mechanisms of miRNAs in plant development, evolution, and adaptation is still limited. This Research Topic presents new and important findings on three fronts: the roles of miRNAs in plant morphology and environmental stresses, the regulatory network based on multi-omics data, and reviews and applications of two classic methods in miRNA study.

Several classical miRNAs are proven to play an important role in plant morphological development. Ren et al., further confirmed this phenomenon in sweet potato by examining the miRNA circuit between miR319 and its target *TCP*s. Intriguingly, they revealed that the significance of *Ib*miR319-targeted *IbTCP*s in leaf anatomical morphology is accredited to photosynthetic rate caused by the change in leaf submicroscopic structure. Branching is another critical morphologic trait in many plants. Mallet et al., first reviewed and highlighted the regulatory contribution and the action of evolutionarily conserved miRNA factors in different species, and then identified seven miRNAs and their nine corresponding target genes potentially involved in branching in Rosebush.

From the early days of studying miRNAs, scientists have found several miRNAs that were involved in the response to environmental stresses in model plants. Li et al., expanded this line of investigation by examining the mechanism of miRNA-mediated root growth and development in response to nutrient deficiency in peanut (*Arachis hypogaea* L.). Under both nitrogen and potassium deficiency, the dry weight of both shoot and root tissues was significantly reduced. Li et al., identified the down- and up-regulated miRNAs (miR156, miR167, miR393, etc.) that were potentially associated with these changes by screening expression patterns of the miRNA target genes under these stress treatments. Villalba-Bermell et al., performed experiments that mimicked the climate change by inducing combined adverse environmental conditions to scan how the core miRNA network reacts. Under the various combinations of four stresses (cold, salinity, short day, and infection with a fungus), they found that a high proportion of the miRNA families showed a non-additive response to multiple stresses in comparison to that observed under each one of the stresses individually.

A recent hotspot of small RNA (sRNA) studies is to examine their roles in responding to diseases caused by bacteria or fungi. Zhang et al., revealed a novel scenario in which *MIR157d* became the target of *Verticillium dahlia*. They showed that a trans-kingdom fungal sRNA, VdrsR-1, could be secreted into host cells and target miR157d and further modulate plant floral transition by affecting the *miR157d/SPL13A/B* regulatory module, resulting in prolonged host vegetative growth that would benefit fungal propagation. On the other hand, our knowledge on the highly conserved miRNAs is expanding due to more inquiries into the functions of their target genes. Taken miR160 and their target *ARF*s (Auxin response factors) as an example, in addition to the classical role in auxin response, functions of the circuits in many aspects of development such as flowering time, fiber length, germination, tillering, leaf morphology, etc., were also reported in different plants. In addition, they were shown to take part in a series of biotic and abiotic stresses, including responses to stress caused by bacteria, fungi and pest, and induced by heat, cold, and drought (Hao et al.).

As multi-omics has become a powerful tool to uncover gene functions, the high noise often hinders its application, especially in non-model plants. This Research Topic features the reporting of two genome scaled miRNA regulatory networks involving upstream transcription factors and downstream targets constructed in lettuce (*Lactuca sativa*, Deng, Qin et al.) and foxtail millet (*Setaria italic*, Deng, Zhang et al.), respectively. By integrating multiple omics datasets including genome, transcriptome, miRNAome, degradome, and the correlation among them, much noise has been removed from the gene regulatory network centered on miRNAs. Consequently, clearer network motifs such as feed-forward loops were identified, and many regulations were discovered and found to be conserved in other species. Therefore, many previously unknown regulations uncovered by these studies are thought to be legitimate for follow-up studies.

Molecular biological and high-throughput sequencing methods for miRNA research have greatly accelerated the understanding of miRNA functions. For instance, the short tandem target mimic (STTM) approach could capture the endogenous miRNAs as a sponge and reveal the effect of miRNA loss-of-functions. Chen et al., reviewed the development and advance of STTM-based methods in plant research, especially in the model crop rice, and discussed the challenges and potential opportunities of combining STTM and CRISPR technology for crop improvement. Another classical method for high-throughput validation of miRNA targets is Parallel Amplification of RNA Ends sequencing (PARE-Seq). Li and Ren creatively applied this method by using publicly available PARE datasets to systematically explore the miRNA processing modes. Their effort revealed a conserved processing mode existing in four examined plants.

The regulatory roles and mechanisms of miRNAs in plant development, evolution, and adaptation are constantly being discovered and expanded, including new functions of those known and conserved miRNAs, even miRNA themselves have become the target of plant-fungus interaction. At the same time, the methods on miRNA research are also continuously evolving. Expanded use of STTM and PARE-seq are providing strong support for functional studies of miRNAs in non-model plants. It has become very promising that the full mining of multi-omics data centered on miRNAs will greatly improve the existing understanding of miRNA function, evolution, etc., in the future. Functional analysis of miRNAs *via* CRISPR-based genome editing as an emerging technology will also greatly value our knowledge of plant miRNAs. miR-CRISPR approach allows us to concurrently knock out miRNA family loci or selectively knock out individual members. Similar approaches can be applied to the target genes of miRNAs. A sophisticated combination of the strategies will provide clearer pictures of the roles of corresponding plant miRNAs in near future.

## Author contributions

XY prepared the first draft of this Editorial. LL, TU, and XZ revised it. All authors approved the Editorial for publication.

## Funding

This work was supported by the National Natural Science Foundation of China (32070248) and Beijing Academy of Agriculture and Forestry Sciences (KJCX201907-2, KJCX20200204, JKZX202201, and KJCX20220105).

## Conflict of interest

Author TU was employed by Ficus Biotechnology. The remaining authors declare that the research was conducted in the absence of any commercial or financial relationships that could be construed as a potential conflict of interest.

## Publisher's note

All claims expressed in this article are solely those of the authors and do not necessarily represent those of their affiliated organizations, or those of the publisher, the editors and the reviewers. Any product that may be evaluated in this article, or claim that may be made by its manufacturer, is not guaranteed or endorsed by the publisher.

